# The Influence of Roughness on Experimental Fault Mechanical Behavior and Associated Microseismicity

**DOI:** 10.1029/2022JB025113

**Published:** 2022-08-13

**Authors:** Barnaby Fryer, Carolina Giorgetti, François Passelègue, Seyyedmaalek Momeni, Brice Lecampion, Marie Violay

**Affiliations:** ^1^ Laboratory of Experimental Rock Mechanics École Polytechnique Fédérale de Lausanne Lausanne Switzerland; ^2^ Now at Sapienza University of Rome Rome Italy; ^3^ Now at Géoazur Université de Côte d’Azur Valbonne France; ^4^ Geo‐Energy Laboratory École Polytechnique Fédérale de Lausanne Lausanne Switzerland

**Keywords:** fault roughness, biaxial experiment, stress heterogeneity, acoustic emission, fault stability, seismic cycle

## Abstract

Fault surfaces are rough at all scales, and this significantly affects fault‐slip behavior. However, roughness is only occasionally considered experimentally and then often in experiments imposing a low‐slip velocity, corresponding to the initiation stage of the earthquake cycle. Here, the effect of roughness on earthquake nucleation up to runaway slip is investigated through a series of dry load‐stepping biaxial experiments performed on bare rock surfaces with a variety of roughnesses. These laboratory faults reached slip velocities of at least 100 mm/s. Acoustic emissions were located during deformation on bare rock surfaces in a biaxial apparatus during load‐stepping experiments for the first time. Smooth surfaces showed more frequent slip instabilities accompanied by slip bursts and larger stress drops than rough faults. Smooth surfaces reached higher slip velocities and were less inclined to display velocity‐strengthening behavior. The recorded and localized acoustic emissions were characterized by a greater proportion of large‐magnitude events, and therefore likely a higher Gutenberg‐Richter *b*_GR_‐value, for smoother samples, while the cumulative seismic moment was similar for all roughnesses. These experiments shed light on how local microscopic heterogeneity associated with surface topography can influence the macroscopic stability of frictional interfaces and the associated microseismicity. They further provide a laboratory demonstration of roughness' ability to induce stress barriers, which can halt rupture, a phenomenon previously shown numerically.

## Key Points

- Experimental‐fault roughness affects fault stability
- Stress heterogeneity related to roughness inferred to halt seismic rupture on millimetric scale
- All stages of seismic cycle observed across seven‐orders of slip velocity magnitude

## 1 Introduction

An earthquake occurs when rupture propagation and slip develop on fault surfaces, such that the understanding of friction and fault geometry is crucial to the understanding of the mechanics of earthquakes. A fault's reaction to stress perturbations can be characterized in a variety of ways depending on its stability (e.g., experimentally (Spagnuolo et al., 2016) and numerically (Cattania & Segall, 2021; Lapusta et al., 2000)): stage 1, the fault remains locked; stage 2, the fault undergoes slow and stable sliding; stage 3, the fault exhibits short‐lived local instabilities; stage 4, the fault accelerates and runaway seismic slip occurs, often with the activation of dynamic weakening mechanisms. The transitions between three first stages can be described through a combination of Mohr‐Coulomb failure and rate‐and‐state friction laws (Barton, 1976; Dieterich, 1979, 1981; Ruina, 1983), such that the frictional response of the fault is dependent on the slip rate and a state variable which accounts for the evolution of the sliding surface. However, stages 3 and 4 are difficult to explore experimentally (e.g., Spagnuolo et al., 2016; Wu & McLaskey, 2019); granted, there is a significant body of numerical work concerning this topic related to fault complexity (e.g., Cattania & Segall, 2021; Dublanchet et al., 2013; Lapusta et al., 2000). Additionally, how the fault transitions to specifically stage 4 and runaway slip is not entirely understood, and how multiscale asperities may influence the reactivation, stability, and runaway of a seismic fault also remains unclear, with this topic having principally only been investigated either theoretically or numerically (e.g., Cattania & Segall, 2021; Dunham et al., 2011; Fang & Dunham, 2013; Sagy & Lyakhovsky, 2019; Tal et al., 2018). Currently, most frictional experiments either focus on very low slip velocities, corresponding to stages 1–3, or on high velocity, corresponding to stage 4.

While both ends of the slip‐velocity spectrum are being investigated, creating the link between low‐ and high‐velocity experiments and natural earthquakes is complicated for two principle reasons. To begin with, most friction experiments do not involve the propagation of a rupture front and therefore do not entirely recreate the weakening occurring during an earthquake. Second, while the gap is slowly being bridged (e.g., Di Toro et al., 2004; Spagnuolo et al., 2016; Wu & McLaskey, 2019), a continuum in slip rate between low‐ and high‐velocity experiments that encapsulates both nucleation and propagation is lacking. In part, this second issue is largely related to the difficulties associated with intermediate‐velocity earthquake experiments (0.1–10 mm/s). In this sense, there exists a gap in the literature in between low‐velocity and high‐velocity experiments. The investigation of earthquake slip rates during the entire seismic cycle is significant not only in regard to our understanding of natural earthquakes and our ability to assess seismic hazard, but also in relation to the sustainable development of anthropogenic activities.

In laboratory experiments, which can reproduce the entire seismic cycle under pressure and temperature conditions representative of the subsurface but in a more controlled manner and on a much shorter time scale than in nature, these investigations typically employ either gouge layers (e.g., Leeman et al., 2016) or bare surfaces without a systematic variation of surface geometry (e.g., Okubo & Dieterich, 1984). However, fault surfaces can be highly heterogeneous, not just in their composition, but also in their surface topographies (Brodsky et al., 2016; Candela et al., 2012). This surface topography, or surface roughness, is present at all scales and a fractal property across nine orders of magnitude (Candela et al., 2012). Roughness changes not just in space, but also in time with slip (Brodsky et al., 2011; Sagy et al., 2007). This heterogeneity is significant because a laboratory fault's surface roughness influences the mechanics of its slip (Biegel et al., 1992; Harbord et al., 2017; Morad et al., 2022; Ohnaka & Shen, 1999; Okubo & Dieterich, 1984; Tal et al., 2018, 2020), the weakening mechanism (Goldsby & Tullis, 2011), and the spatial distribution of the micro‐seismicity (Goebel et al., 2017), with the implication that fault roughness can influence foreshock and aftershock activity (Aslam & Daub, 2018; Cattania & Segall, 2021; McLaskey & Lockner, 2014). Additionally, on the kilometric scale, fault geometry has been observed to have a significant influence on earthquake nucleation and rupture termination (Aki, 1979; King, 1986; King & Nábělek, 1985; King & Yielding, 1984). Moreover, as roughness acts as a proxy for stress heterogeneity (e.g., Candela et al., 2011; Cattania & Segall, 2021), a better understanding of its influence on earthquake nucleation can also be related to the influence of stress barriers, which have been shown to be capable of halting an already‐nucleated earthquake's propagation along both kilometric‐scale natural‐ (Aki, 1979; Gupta & Scholz, 2000; Husseini et al., 1975; Lay & Kanamori, 1981) and metric‐scale laboratory‐ (Ke et al., 2018) faults. A laboratory demonstration of roughness's ability to impede earthquake nucleation would therefore enlarge the scope of observation of this type of effect to a third, millimetric scale.

By performing load‐controlled experiments in the High Strain TEmperature Pressure Speed (HighSTEPS) apparatus (Violay et al., 2021), a low to high velocity biaxial friction apparatus located at the EPFL in Switzerland, it has been possible to investigate the transition from velocity‐strengthening to velocity‐weakening behavior. Load‐stepping in biaxial apparatuses is rarely employed, barring a few examples using gouge material (e.g., Dieterich, 1981; Scuderi et al., 2017), but this approach allows for a development of slip which is spontaneous and a loading which is more readily compared to natural seismicity, where the far‐field stress together with the frictional properties of the fault govern the behavior of the fault. The experiments performed here transverse the low velocities frequently investigated in rate‐and‐state friction (μm/s) and surpass the intermediate velocities (reaching up to 100 mm/s) for which there is generally a dearth of data in experiments that include a nucleation phase. In particular, these experiments will focus on the influence of roughness on the transition from a locked fault to slow and controlled to then fast and runaway slip, covering all four phases of the seismic cycle. Through the analysis of acoustic emissions and the use of load‐stepping such that the slip velocity is allowed to freely evolve, it will be possible to provide experimental evidence to the previously numerically developed notion (Cattania & Segall, 2021) that stress heterogeneity associated with roughness can halt or prevent dynamic rupture along rough faults, with relative stress homogeneity along smooth faults leading to their more complete and dynamic rupture.

## 2 Experimental Methodology

Experiments were performed on three different roughnesses with the same stress boundary conditions, with a second set of experiments to demonstrate reproducibility (see Supporting Information S1).

### 2.1 Sample Preparation

All six experiments concerned bare rock surfaces of Galaxy Noir norite. Galaxy Noir, also known commercially as Star‐ or Black‐Galaxy Granite, is a norite from the Ongole quarry in southern India. This intrusive mafic igneous rock is comprised of dark plagioclase feldspar and bright bronzite pyroxene crystals. The dry density of Galaxy Noir was calculated based on a sample's dimensions and weight as 2,950 kg/m^3^. This rock has been chosen for its homogeneity and small (typically less than 2 mm) grain size, making it ideal for experimental investigations.

**Figure 1:**
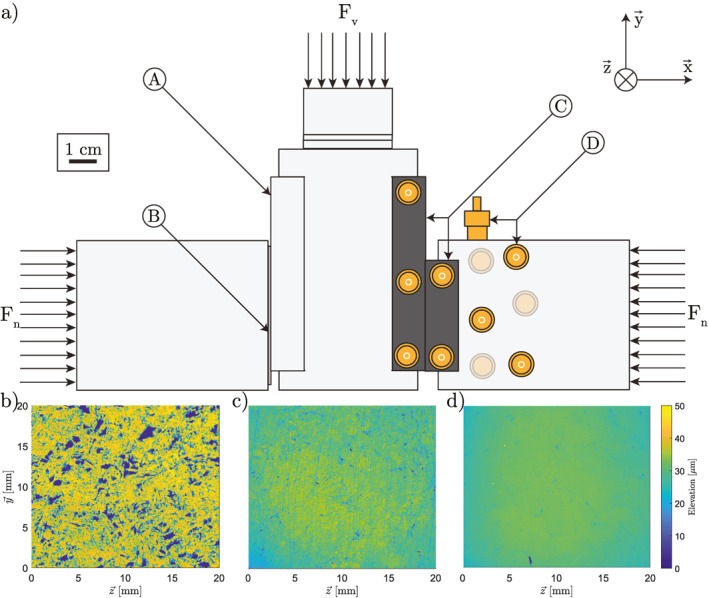
(a) Side‐on view of biaxial arrangement used during the experiments. (A) Stainless steel block, (B) near‐frictionless piece composed of GLYCODUR, which, with (A), comprises a near‐frictionless surface, (C) sample, (D) acoustic sensors and their approximate positions, faded sensors are located on the backside of the setup. The normal force is applied by a horizontal piston on the right hand side of this image. The left hand side of the setup is supported by the internal wall of the HighSTEPS apparatus. The vertical (shear) load is applied by the vertical piston through the central sample holder. The sample holders and accompanying unlabeled pieces are composed of stainless steel. (b–d) Optical interferometry microscope scans of a portion of the prepared sample surfaces prior to their use during an experiment. (b) The roughest sample, presenting an *R*_a_ of 14.44 μm and an *R*_q_ of 21.87 μm, prepared using the milling cutter. (c) The sample of medium roughness, presenting an *R*_a_ of 3.15 μm and an *R*_q_ of 4.51 μm, prepared with an 80‐grit grinding disc. (d) The smoothest sample, presenting an *R*_a_ of 1.58 μm and an *R*_q_ of 2.23 μm, prepared with a 1,200‐grit grinding disc. *R*_a_ and *R*_q_ measurements were taken across a surface area of 20 × 20 mm. As discussed by previous authors (Brown & Scholz, 1985), the hand polishing of smooth samples does lead to a slight dome‐up shape.

Samples were cut out of plates of Galaxy Noir into rectangular prisms of size 110 × 35 × 12 mm. The faces were then ground flat (±100 μm precision), with tap water used for cooling. At this stage, the roughness was applied to the samples using either Struers resin‐bonded diamond grinding discs or a milling machine. The roughness of the two smoothest samples was applied by first using an 80‐grit grinding disc and then a 1,200‐grit grinding disc. The two medium roughness samples were prepared just using the 80‐grit grinding disc. The grinding discs were always used with tap water wetting the disc, and a figure‐8 pattern of movement was used when applying the roughness by hand. The two rougher samples' surfaces were prepared using an OPTImill MH 25SV milling machine and a 63‐mm PK 63‐10 E milling cutter equipped with two DIXI 26420 APKT 10.03.05 PCD SP diamond inserts. The samples were simply passed under the milling cutter at 1,000 rpm and 2.5 mm/s with tap water as a cooling fluid. The roughnesses of the surfaces were then measured using a Bruker Contour GT‐K 3D Optical Profiler (Figure 1). The root mean squared roughnesses of the surfaces was then calculated using an in‐house MATLAB script (see repository file) across a sample area of 20 mm by 20 mm. The two roughest samples presented an arithmetic mean deviation, *R*_a_, of 10.70 and 14.44 μm and a root mean square roughness, *R*_q_, of 16.54 and 21.87 μm. The two medium roughness samples presented an *R*_a_, of 3.15 and 3.09 μm and an *R*_q_, of 4.51 and 4.60 μm. The two smoothest samples presented an *R*_a_, of 1.58 and 1.91 μm and an *R*_q_, of 2.23 and 2.69 μm. Additionally, using the FSAT software of Heinze et al. (2021), the average Hurst exponents of the principal rough, medium, and smooth samples were found to be 0.44, 0.38, and 0.60 in the direction of slip, respectively. Further roughness measurements are provided in the Supporting Information S1. Note that the intention is not to reproduce the roughness of natural faults but to systematically investigate its effects.

The 110‐mm length samples were then cut into two pieces of 70 and 40 mm in length, yielding samples which were 70 × 35 × 12 mm and 40 × 35 × 12 mm. The newly cut edges were then ground flat, with tap water used for cooling. Pre‐experiment photos of all samples were taken at this stage (see Supporting Information S1).

### 2.2 Load‐Controlled Biaxial Experiments

#### 2.2.1 Apparatus

Biaxial load‐controlled experiments were performed in the HighSTEPS (Violay et al., 2021) apparatus (Figure 1). The horizontal piston can apply a normal load of up to 160 kN. The vertical piston can apply a maximum shear load of 193 kN and achieve shear velocities of 0.25 m/s and accelerations of 10 m/s^2^. Note that the optical encoder on the vertical piston is used for the vertical position measurements and corrected only for the stiffness between the optical encoder and the sample, meaning the velocities reported will represent minimum values. While the apparatus is set up in double‐direct shear, on one side of the sample holder a near‐frictionless GLYCODUR piece was placed in contact with a stainless steel block, creating a near‐frictionless surface. Performing the experiments in single‐direct shear helped ensure that all samples had the same roughness and that good contact between the samples was achieved, while also facilitating the localization of the acoustic emissions.

Twelve P‐wave acoustic sensors were glued to the sample and sample holder, with five placed directly on the sample and seven on the sample holder (Figure 1). Each sensor was composed of a PZT crystal contained in brass casing. Passively recorded acoustic emissions were recorded with a sampling rate of 10 MHz and amplified at 35 dB through pre‐amplifiers. Amplified signals were recorded if at least five sensors recorded an amplitude greater than 0.15 V.

### 2.3 Experimental Procedure

The samples were placed in the sample holders with ultrasonic couplant and mounted into the HighSTEPS apparatus. A normal force, *F*_n_, of 2 kN was applied and held for 30 min before being increased to a load corresponding to 20 MPa, considering the sample's dimensions. This higher load was also held for 30 min, at which point a run in at 1 μm/s was performed until reaching a vertical force, *F*_v_, of 14 kN, corresponding to a ratio of $\frac{F_{v}}{F_{n}}$ of approximately 0.5 for most samples. This methodology prepared the sample surface and ensured good contact without applying a large shear stress. At this stage the vertical load was reduced such that $\frac{F_{v}}{F_{n}}$ was equal to 0.4. These conditions were held constant for 1,000 s, at which point the vertical load was increased such that $\frac{\Delta F_{v}}{F_{n}}=0.02$. This step‐wise increase of vertical load was repeated until runaway rupture was achieved. The mechanical data were recorded at a recording frequency of 200 Hz while $\frac{F_{v}}{F_{n}}$ was between 0.42 and 0.58, 500 Hz while $\frac{F_{v}}{F_{n}}$ was between 0.60 and 0.68, and 2 kHz while $\frac{F_{v}}{F_{n}}$ was 0.70 or greater.

Once a slip of 35 mm had been achieved, the sample was unloaded first in the vertical and then in the horizontal directions. The samples were then photographed, scanned with an optical profiler, and stored.

## 3 Results

### 3.1 Shear Stress and Slip

**Figure 2:**
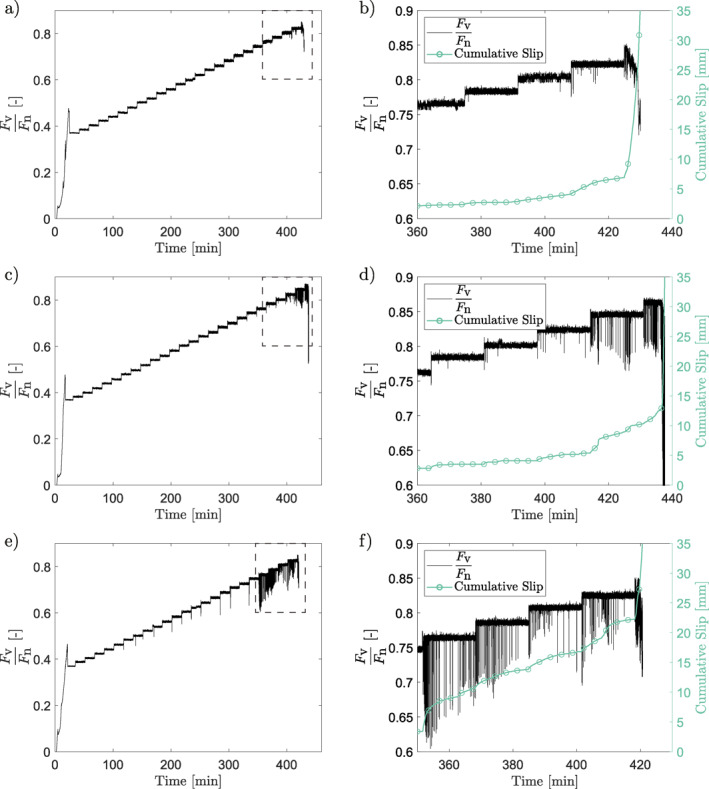
(a and b) The roughest sample tested, (c and d) the sample of medium roughness, and (e and f) the smoothest sample. (a, c, and e) An overview of the development of the ratio between vertical and horizontal force on the sample throughout the experiment. The 1,000‐s‐long vertical force holds can be clearly seen. The stress drops can be seen to be larger and more frequent the smoother the sample is. The dashed box shows the location of the zoom for (b, d, and f), which more clearly show the force ratio and slip for the last few steps. The stress drops can be seen to be larger the smoother the sample is. The smoothest sample achieved significantly more slip in the steps prior to the final one.

**Figure 3:**
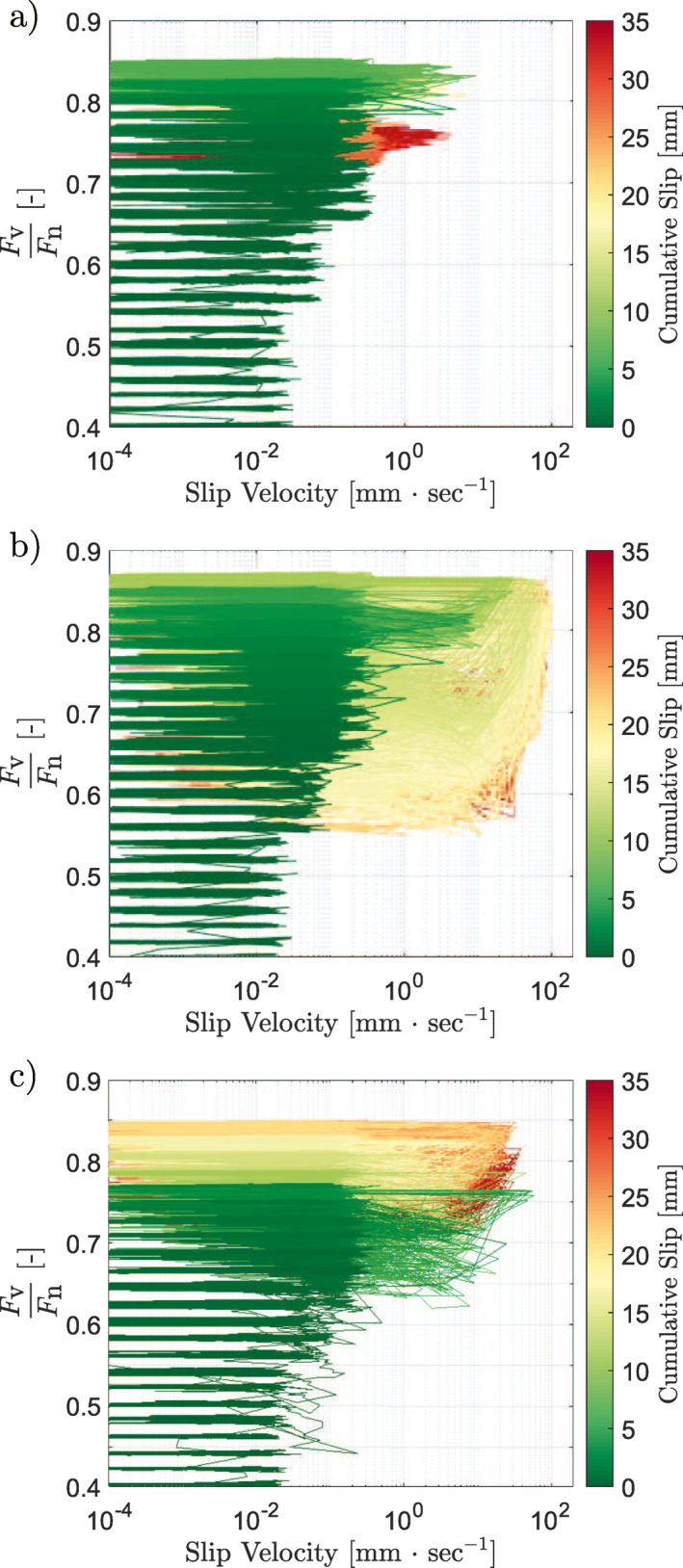
The shear velocity development as the ratio between vertical and horizontal force was increased. The accumulated slip at a given point in the experiment is shown by the color bar. (a) The roughest sample tested, (b) the sample of medium roughness, and (c) the smoothest sample. The stress drops can be seen to be larger, and the velocity achieved greater, the smoother the sample is. The smoothest sample also achieved significantly more slip in the steps prior to the final one. Note that the high‐velocity slip occurring in (b) is principally occurring during the catastrophic failure at the end of the experiment.

**Figure 4:**
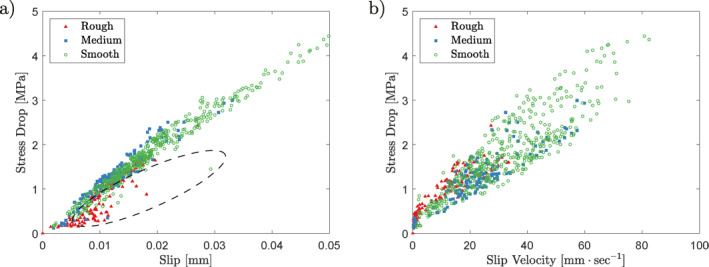
(a) The stress drop as a function of the slip occurring during a stress‐drop event. Note that most stress drops follow a single trend, but that certain points fall below this trend (circled). This will be discussed to be related to whether or not the entire sample surface slips in a later section. (b) The magnitude of stress drop versus the maximum slip velocity achieved during that stress drop. All six experiments are represented in the plot. The values are taken when the shear stress departs from its set value by at least 200 N. Only instances where the stress recovers to its set value are used. The stress drop data are picked automatically and then controlled manually, see Supporting Information S1. The stress drop is taken as the difference between the set and minimum shear stress of the event. The velocity of the stress drop is taken as the maximum velocity between the start of the stress drop and the minimum value of shear stress. The slip is taken as the amount of displacement between the initiation of the stress drop and the recovery of the stress.

The load‐stepping experiments were performed to characterize the behavior of bare norite surfaces with varying roughnesses as the surfaces transitioned from a locked to a catastrophically slipping behavior. The mechanical results (Figures 2, 3, 4) show initially stable behavior in all cases, independent of initial roughness. As loading progresses, in some instances short‐lived slip events occur at an increase in loading. Additionally, the slip events become more regular, occurring spontaneously throughout a loading step. At a certain load, the samples are unable to support the shear load and runaway slip occurs. While this behavior is an accurate description of the global behavior of all of the samples, there is a clear difference for different surface roughnesses. Smoother samples exhibit slip events beginning earlier in the loading (at $\frac{F_{v}}{F_{n}}=$ 0.82 and 0.86 for the rough samples, 0.80 and 0.82 for the medium samples, and 0.78 and 0.82 for the smooth samples), achieving more total slip prior to catastrophic failure (7 and 6 mm for the rough samples, 10 and 5 mm for the medium samples, and 22 and 17 mm for the smooth samples). These slip events are additionally more frequent and reach higher velocities (up to 35 mm/s for rough samples, 60 mm/s for medium samples, and 80 mm/s for smooth samples) and total amount of slip per slip event (up to 20 μm for rough samples, 32 μm for medium samples, and 50 μm for smooth samples) (Figure 4) for smoother samples. The total amount of slip that occurred during the stress drop events across the entirety of the experiments is also larger for the smooth samples (0.28 and 1.29 mm for the rough samples, 1.17 and 0.63 mm for the medium samples, and 3.37 and 4.83 mm for the smooth samples). The stress drops that accompany these slip events are also larger for smoother samples. Finally, note that, while both the medium and smooth samples achieve velocities above 50 mm/s, these velocities only occur after the initiation of runaway slip in the medium sample, whereas the smooth sample achieves them while being intermittently stable. Generally, the relationship in Figure 4a between stress drop and slip follows one linear trend; however, there are a number of smaller events which present low values of stress drop for a given value of slip.

In each case, the initiation of the run‐in phase is accompanied by a shortening of the samples (see Supporting Information S1). During this run‐in phase, the sample switches from a compacting to a dilating behavior. This dilating behavior continues for the rest of the experiment, accelerating near the end when the sample begins to slip catastrophically. This dilatancy is most probably associated with either gouge formation and/or misalignment. Notably, the post‐mortem samples all present significant amounts of material build‐up on their surfaces (see Supporting Information S1 for photos).

### 3.2 Acoustic Data

Acoustic emissions are located using a semi‐automatic and parallel algorithm presented by Momeni et al. (2021). Before localization, the signals are pre‐processed, and high‐ and low‐frequency noise is removed. Then, the hyperparameters of the localization algorithm are tuned based on the design of the passive network and the quality of the signals. For sensors located on the steel body of the loading system, the effect of ray refraction due to the velocity change from rock to steel is corrected for. Also, the contributions of each medium to the P‐wave travel times of the acoustic‐emission signals are included in the localization process. Further details are provided in the Supporting Information S1.

Taking acoustic emissions that have an rms‐location accuracy of less than 1.5 μs, using at least six sensors, and with an azimuthal gap of less than 180°, the relative magnitudes, *M*_R_, of the events were estimated using the method presented by Zang et al. (1998). Prior to relative magnitude estimation, the effect of pre‐amplifier gains are removed from the signals. The relative magnitudes were used to find a proxy for the seismic moment, $M_{0}^{*}$,

(1) $$M_{0}^{*}=10^{\frac{3}{2}\left(M_{R}+10.7\right)}.$$

**Figure 5:**
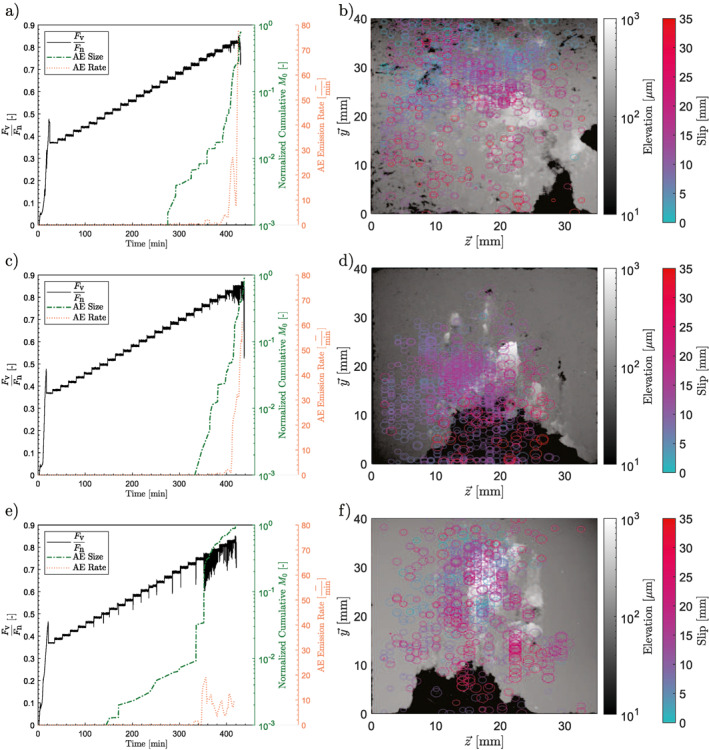
(a, c, and e) Events located with a rms accuracy of 1.5 μs or less, the normalized cumulative seismic moments, and emission rates of the experiments. The normalized cumulative seismic moment is calculated based on a conversion of the relative magnitudes to seismic moments and then a normalization based on the largest total seismic moment from the three tests. The development of the ratio between vertical and horizontal force on the sample throughout the experiment, as seen in Figure 2, is also plotted. (b, d, and f) Optical profiler interferometry scans of the post‐mortem sample surfaces with the developed gouge still present on the sample surface. Overlain with colored circles are the located events, with their color corresponding to the amount of slip at the time of the event. The size of the circles corresponds to the size of the event, such that a plotted circle diameter of 0 mm corresponds to a relative magnitude of −3.5 and a plotted circle diameter of 2 mm corresponds to a relative magnitude of −0.8, with the diameter scaling linearly between these two values. (a and b) The roughest sample tested, (c and d) the sample of medium roughness, and (e and f) the smoothest sample.

Note that these are not true seismic moments and can only be used to compare within this set of experiments. The normalized cumulative acoustic emission size as well as the acoustic emission rate were plotted for each experiment (Figures 5a, 5c and 5e). The cumulative seismic moment, found by summing the $M_{0}^{*}$ of all of the acoustic emissions for each experiment, was similar for all three experiments (Figures 5a, 5c and 5e), but was larger for smoother samples.

Post‐mortem optical profiler interferometry scans were taken of the samples (Figures 5b, 5d and 5f). These scans were overlain with acoustic emissions located on the fault plane. The smoother the sample the more concentrated and larger the gouge buildup observed upon visual inspection. Note that the acoustic emissions for the smooth sample occur earlier in time, but there is no clear trend when plotted as a function of cumulative slip. Upon visual inspection, smoother samples exhibit more large‐magnitude events than rougher samples, with these events being more spatially concentrated, especially in areas of gouge buildup.

**Figure 6:**
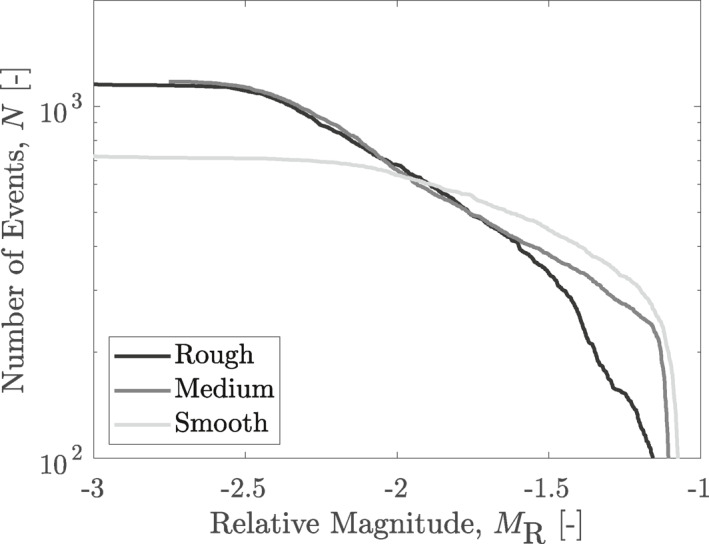
The frequency‐magnitude distributions of all three roughnesses taken from the acoustic emissions data, with the lighter lines depicting smoother samples.

The frequency‐magnitude distributions in Figure 6 show less small events and more large events for smoother samples. While the number of acoustic emissions and magnitude range traversed by the experiments are not sufficient to be fit in a Gutenberg‐Richter framework, qualitatively it can be seen that smooth samples would likely exhibit a lower Gutenberg‐Richter *b*_GR_ exponent. Further support for this trend can be seen in the increased slip displacements during stress drops observed for smoother samples (Figure 4a).

## 4 Discussion

### 4.1 Macroscopic Fault Behavior

#### 4.1.1 Stability

**Figure 7:**
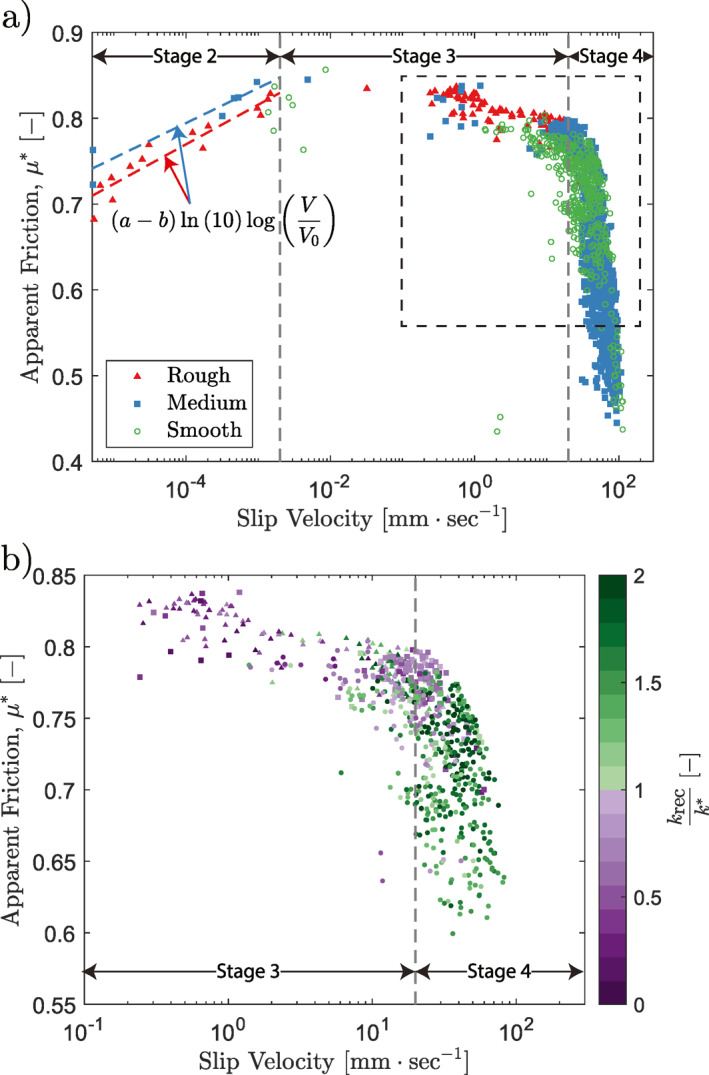
(a) The apparent friction versus slip velocity for the three different surface roughnesses. Each data set begins with a period of elastic loading (stage 1), not shown as slip velocity is zero. This is followed by a period of low‐velocity slip (stage 2). In the case of velocity‐strengthening behavior, μ* increases linearly with the logarithm of slip velocity and can be fit to find values of $\left(a-b\right)$ for the rough and intermediate roughnesses (0.02 and 0.018, respectively). Then, samples exhibiting velocity‐strengthening transition to velocity weakening and short‐lived instabilities are observed for all samples (stage 3). Finally, stress drops become larger and catastrophic failure begins, often associated with the activation of a dynamic weakening mechanism (stage 4). The approximate boundaries of the stages are delineated. All six experiments are plotted. (b) A zoom on the section of (a) delineated by the dotted square. The color bar represents the ratio of the stiffness taken from the recovery phase of the stress drop to the unloading stiffness at the end of the experiment. Only stress drop events which recover to the set stress are used.

To assess the stability of the rough and smooth faults, the apparent friction, μ*, or the ratio of vertical to horizontal force during a slip event, versus slip velocity is plotted using the steady‐state slip velocity across each of the initial stable shear‐stress steps and the slip velocities measured during the stress drop events at the end of the experiments, such that seven orders of magnitude of slip velocity and all four stages of the seismic cycle are traversed (Figure 7). The data agree in form with the results presented by Spagnuolo et al. (2016). Note that the rougher samples have significantly more low‐velocity data points than the smooth samples. This is because the rough samples have a larger tendency to slip stably at low velocity, whereas the smooth samples are either almost entirely locked or slipping dynamically, as shown previously in numerical simulations (Cattania & Segall, 2021; Tal et al., 2018). All data points above 10 μm/s are taken from the dynamic stress drop data; all data points below this value are steady‐state slip velocities.

The slip velocities for the initial, pre‐dynamic stress‐drop velocities of this analysis are found by linearly fitting the slip as a function of time for each step, with examples of these fits given in the Supporting Information S1.

Fault reactivation, or the departure from stage 1 as governed by Mohr‐Coulomb failure criterion, occurs at lower values of friction for rough samples as compared to smooth samples, in agreement with previous findings (Biegel et al., 1992; Marone, 1998). This is likely due to rougher faults having patches of low normal stress which therefore have lower frictional strength (Cattania & Segall, 2021); or more generally patches with a lower stress criticality. The transition to stage 3, seems to occur at higher values of macroscopic apparent friction for rough samples.

On a macroscopic scale, once slip has initiated at the end of stage 1, two related conditions are necessary for unstable slip and the ultimate passage to stages 3 and 4 within the RSF theoretical framework: one requiring velocity‐weakening friction (Rice & Ruina, 1983; Ruina, 1983), and another associated with the stiffness of the fault and the surrounding medium (Dieterich, 1979).

The first of these two conditions is typically presented in terms of the direct and evolution effect, *a* and *b* respectively, and specifically their difference, which represents the condition necessary for unstable slip (Ruina, 1983). The second necessary condition states that for unstable slip the rate of elastic unloading must be exceeded by the weakening rate of the fault (e.g., Dieterich, 1979; Marone, 1998). In the case that slip rates are subseismic (less than 1 $\frac{cm}{\sec\;}$) and can be modeled by rate‐and‐state friction, the frictional properties of the fault are given in terms of a critical stiffness, *k*_c_, such that slip can become unstable when the system stiffness, *k*, falls below *k*_c_ (Dieterich, 1979; Rice & Ruina, 1983) or when the system is submitted to a large velocity kick (Gu et al., 1984). This critical stiffness can be approximated by the combined machine‐sample stiffness, k*, which is found via the second unloading stiffness at the end of the experiment (see Supporting Information S1 for details).

As can be seen in Figure 7b, the transition from stage 3 to stage 4 occurs when the fault system reaches this critical stiffness (i.e., when the global fault stiffness measured during the recovery phase of the stress drop, *k*_rec_, exceeds the stiffness of the system, k*). Prior to this, in stage 3, the fault is exhibiting velocity‐weakening behavior but only a section of the fault ruptures; this section of the fault may be locally stiff enough for seismic slip; however, only when the system stiffness is smaller than the macroscopic critical (fault) stiffness can the entire fault rupture. Based on this interpretation, the distribution of stress drop events for each set of experiments implies a higher fault (critical) stiffness for smooth faults and a higher proportion of contained ruptures for rougher samples. An alternative explanation would involve an increased system stiffness for rough surfaces, as previously suggested (Tal et al., 2020); however, the system‐unloading stiffnesses found here cannot account for the observed differences (63.7 and 71.9 kN/mm for the rough samples, 122.9 and 108.7 kN/mm for the medium samples, and 87.1 and 91.4 kN/mm for the smooth samples). Finally, that reaching a critical stiffness corresponds to the rupturing of the entire sample surface implies a scale dependence for dynamic rupture.

The second criterion for stability can also be addressed in terms of the critical length, *L*_c_ (Okubo & Dieterich, 1984). The critical length is defined as the length of the slipping region up to which stable sliding occurs in a nucleation phase. Beyond this length the slip front becomes unstable and accelerates toward shear wave speed. The critical length is defined as (Ruina, 1983),

(2) $$L_{c}=\frac{ED_{c}}{2\left(1-\nu^{2}\right)\sigma_{n}\left(b-a\right)},$$

where *σ*_n_ is the effective normal stress, *D*_c_ is the characteristic slip or slip weakening distance, *E* is Young's modulus, and *ν* is Poisson's ratio. Considering the increased instability for smooth surfaces seen here, observable for example, via the larger size of stress drops (Figures 2, 3, 4), the velocity‐strengthening behavior seen in stage 2 for rougher samples (Figure 7), and the minimum size of recorded acoustic emissions (Figure 6), the implication is that smoother fault surfaces exhibit a smaller critical length, as shown previously (Ohnaka, 1996; Ohnaka & Shen, 1999; Okubo & Dieterich, 1984). Indeed, the slip weakening distance decreases with decreasing roughness (Dieterich, 1979, 1981; Ohnaka & Shen, 1999; Okubo & Dieterich, 1984). Additionally, low‐velocity friction experiments have indicated that $\left(b-a\right)$ is larger for smoother faults (Dieterich, 1981; Harbord et al., 2017). This is further supported by these experiments, where $\left(a-b\right)$ in stage 2 was found to be 0.02 for rough samples, 0.018 for intermediate roughnesses, and velocity weakening for smooth samples. Note that in stage 3, all samples transition to a velocity‐weakening behavior. Interestingly, Figure 7 admits two values of velocity corresponding to a single‐stress state. Therefore, an experimental fault expresses a dual nature that might explain the rise of marginally stable slip pulses (stage 3) in relatively high‐stress states.

Finally, the stress drops seen in the smoother samples are larger and account for more slip than in rougher samples, in agreement with previous observations (Dresen et al., 2020). While the smoother faults exhibited stress drops earlier on in the loading phase than the rougher faults, the majority of the energy release was more sudden for the smooth faults than the rough ones (Figures 5a, 5c and 5e). This result is also in agreement with the findings of Dresen et al. (2020).

### 4.2 Weakening Mechanism

Considering the slip velocities achieved along the faults (Figure 7), and that these slip velocities represent minimum values as a correction for the sample stiffness has not been applied, it seems likely that flash heating is the active weakening mechanism in these experiments. In particular, flash heating has been shown to be active in gabbro, which is similar to norite in terms of its composition, at approximately these slip velocities (Niemeijer et al., 2011; Passelègue et al., 2014).

As slip progresses, it is possible that samples achieved similar ultimate microstructures, which could explain the similar weakening rates; however, further characterization would be required to confirm this point. Higher slip velocities can lead to the activation of a number of other dynamic weakening mechanisms (Di Toro et al., 2010; Niemeijer et al., 2011). In particular, it is possible that, given the differences in real contact area between smooth and rough samples (Dieterich & Kilgore, 1996; Hisakado, 1974) and their differing propensities for high‐velocity unstable slip, smooth samples may experience the activation, or at least increased relevance, of certain dynamic weakening mechanisms at lower stresses and/or slip velocities (Goldsby & Tullis, 2011; Rice, 2006). This is supported by the recent results of Harbord et al. (2017), showing gouge formed during cataclasis and frictional melting for samples of different roughnesses tested at the same normal stress and the results here, which show a transition to velocity‐weakening at lower shear stresses for smoother samples (Figure 7). Ultimately, however, all the samples here seem to converge to the same weakening mechanism despite their differences in roughness.

### 4.3 Microscopic Fault Behavior

#### 4.3.1 Insights From Acoustic Emissions

Although only qualitative in nature, the Gutenberg‐Richter *b*_GR_ value has been shown here to correlate inversely with roughness (Figure 6), in agreement with the findings of Goebel et al. (2017). Further, while the roll‐off at low magnitudes in the frequency‐size distributions of Figure 6 were considered to be due to catalog incompleteness, it is possible that the higher magnitude of completeness seen for smooth samples could be due to a larger minimal nucleation size of these samples related to a higher correlation length of stress, as suggested previously (Ampuero et al., 2006).

While stage 2 was characterized by velocity‐strengthening behavior, acoustic emissions were recorded during this stage for all experiments (Figure 5). This implies that, at the asperity scale, the samples are actually characterized by velocity‐weakening behavior and that the critical length at this scale is smaller than the size of the asperity. This further implies a scale dependence of velocity‐weakening/strengthening behavior.

#### 4.3.2 Effect of Surface Heterogeneity on Ubiquitous Fault Activation

Interestingly, the cumulative acoustic emission energy released at the end of each of the three experiments is similar, albeit slightly larger for smoother samples, suggesting that rough samples exhibit events which are spread out and that cannot interact with each other, with events that are always smaller than the critical length of the fault and therefore do not lead to its early macroscopic instability. Indeed, a possible interpretation is that a more heterogeneous stress state on rough samples due to large variability in asperity size can act as a stress barrier to these local ruptures. Conversely, local events on smooth faults can influence each other and lead to global instability due to the relative homogeneity of the stress state along the fault. This explanation is based on the stiffness and acoustic‐emission size arguments presented above and is in accordance with previous results from fracture mechanics (Bayart et al., 2016; Freund, 1998; Galis et al., 2017; Kammer et al., 2015; Lebihain et al., 2022) which have shown that the propagation of seismic ruptures depends on the local fracture energy along the interface, itself a function of the initial stress along the fault plane; it has further been supported numerically (Cattania & Segall, 2021). Stress heterogeneity's ability to halt rupture has been further observed in metric‐scale experiments (Ke et al., 2018) and on kilometric‐scale natural faults (Aki, 1979; Gupta & Scholz, 2000; Husseini et al., 1975; Lay & Kanamori, 1981). The observation of this effect is extended to a third, millimetric scale by the demonstration here of roughness's ability to inhibit earthquake nucleation on the tested laboratory faults combined with roughness's previously demonstrated correlation with stress heterogeneity (e.g., Candela et al., 2011; Cattania & Segall, 2021). Further, this implies that roughness at larger scales (Candela et al., 2012) may also have a significant influence on the slip mechanics of large faults, as previously observed (Aki, 1979; King, 1986; King & Nábělek, 1985; King & Yielding, 1984).

**Figure 8:**
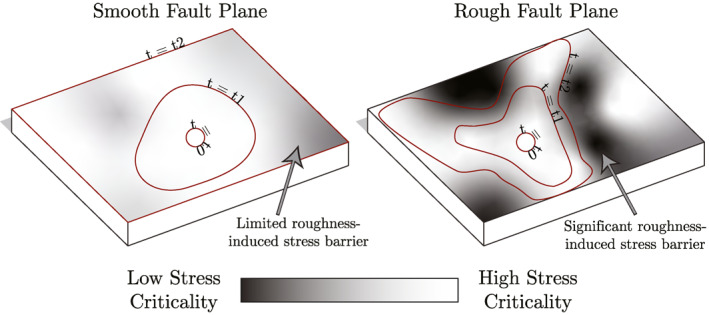
An example schematic illustrating how roughness‐induced stress heterogeneity, which results in heterogeneous apparent friction, is proposed to lead to a propagating rupture being halted, such that it is unable to induce slip across the entire sample surface. Smoother surfaces, which have less geometric heterogeneity along their surfaces, are more prone to having their entire surface ruptured, resulting in higher‐velocity, dynamic slip events. Note that a slip event would allow for the redistribution of stress along the sample surface, meaning that other areas of the sample may be able to rupture during subsequent slip events. The qualitative stress criticality is shown by the black and white color bar, with white corresponding to critically stressed. Stress criticality here refers to ratio of vertical to horizontal stress active at a given location compared to the apparent friction required to activate slip at that same location. Smooth surfaces are interpreted to have more limited roughness‐induced stress barriers than rough surfaces, which are characterized by roughness‐induced stress barriers which are larger and more significant for rupture propagation. The rupture front of each case is shown at three points in time, *t* = *t*_0_ < *t*_1_ < *t*_2_, with *t* = *t*_0_ corresponding to nucleation.

This mechanism, and the more significant amount of supposed stress heterogeneity in the rough samples, can be used to explain the larger proportion of low magnitude events for rough samples, where events that nucleate on rough samples are more likely to be halted by a stress barrier on a neighboring asperity leading to smaller events (Figure 8). Further, it can explain the low steady‐state slipping velocities seen in Figure 7 on rough samples, as it implies that rough samples are able to slip locally on the fault surface, without causing the entire fault surface to slip. Slip on smooth samples, on the other hand, is more likely to lead to the entirety of the fault surface slipping, resulting in higher‐velocity, dynamic slip events.

Previously, Goebel et al. (2017) highlighted acoustic emissions becoming more spatially distributed with increasing roughness. In contrast, Dresen et al. (2020) recently demonstrated increased clustering for rougher samples. In this suite of experiments, there are visual indications that the events become more spread out across the sample surface with increasing roughness. This trend is coincident with the increased spreading of gouge formation seen on the rougher samples (Figures 5b, 5d and 5f).

## 5 Conclusion

In order to study the effect of roughness on fault behavior, load‐stepping biaxial experiments on bare surfaces with systematically varied roughnesses were performed, traversing seven orders of slip velocity beginning in a locked state, unlocking with slow and stable slip, and finally achieving runaway slip. In these experiments, smooth surfaces exhibited more unstable behavior than rough ones. This is likely related to the smaller critical lengths of smooth fault surfaces and the reduced number of stress heterogeneities that can act as a barrier to earthquake nucleation, with these experiments acting as evidence of a stress barrier's ability to halt dynamic rupture on a laboratory or asperity scale. The smooth samples displayed more velocity‐weakening behavior than rough samples and exhibited qualitatively lower Gutenberg‐Richter *b*_GR_ values, with a higher localization of acoustic emissions and gouge formation. Finally, however, these surfaces of differing roughnesses converged to the same weakening profile and mechanism. By performing the experiments in load‐stepping as opposed to the typical low‐velocity rate‐and‐state friction experiments, the influence of roughness was framed in a way more related to natural earthquakes, from fault loading to earthquake nucleation to runaway slip.

## Supporting information

Supporting Information S1

## Data Availability

The collected data and the MATLAB functions/scripts used to evaluate the mechanical, acoustic, and roughness data have been made available online and can equivalently be requested from the corresponding author (Fryer et al., 2021).
