# Supplementary material for: The Influence of Roughness on Experimental Fault Mechanical Behavior and Associated Microseismicity
Source: J Geophys Res Solid Earth. 2022 Aug 13;127(8):e2022JB025113. doi: 10.1029/2022JB025113 (PMC9539693; doi:10.1029/2022JB025113)
Supplement: Supplementary file 1 — Supporting Information S1 [file JGRB-127-e2022JB025113-s001.pdf]

Supporting Information for "The influence of roughness on experimental fault mechanical behaviour and associated microseismicity"

Barnaby Fryer[1], Carolina Giorgetti[1], François Passelègue[1], Seyyedmaalek Momeni[2], Brice Lecampion[2], Marie Violay[1]

[1]Laboratory of Experimental Rock Mechanics, École Polytechnique Fédérale de Lausanne, Station 18, 1015 Lausanne, Switzerland

[2]Geo-Energy Laboratory, École Polytechnique Fédérale de Lausanne, Station 18, 1015 Lausanne, Switzerland

General Data treatment and Analysis

The vertical and horizontal displacements measured by the optical encoders on the vertical and horizontal pistons, respectively, were corrected for the elastic distortion of the apparatus, following the machine stiffnesses reported in Violay et al. (2021) with a slight adjustment for the vertical displacement of the optical encoder, which was corrected with a machine stiffness of 199 $\frac{\mathrm{kN}}{\mathrm{mm}}$ up to 5 kN and 1018 $\frac{\mathrm{kN}}{\mathrm{mm}}$ above 5 kN. The vertical load was then further corrected for the contribution of the near-frictionless surface, Figure 1 (main text), which was found from calibration testing to support 0.4 kN of the applied load at 20 MPa normal stress. The slip velocity was found simply from the corrected vertical displacement and the recording frequency. Other than the horizontal load, the mechanical data were not filtered. The proportional gain, integral, and derivative settings on the horizontal piston's PID were 0.003, 0.02, and 0.001, respectively.

The Picking of Stress Drops

Stress drops are picked automatically and then checked by hand. When the vertical force departs from its set value by a given amount, a point a given distance behind this point is chosen as the start of the stress drop. The minimum value of vertical force after this point is taken to calculate the value of the stress drop. The velocity of the stress drop is taken between the initiation of the stress drop and this minimum. When the vertical force recovers to its initial value, the slip accrued during the stress drop is calculated, Figure S1. The specific values used to automatically pick are set by manually checking the success of the algorithm with those set values. Each stress drop is always checked manually to make sure it is reasonable.

The Calculation of Stiffnesses

The stiffness of the entire system (machine and sample in series) was taken to represent k^*. This value was found from the second unloading stiffness as shown in Figure S2.

The recovery stiffness of each stress drop, k_{rec}, is found by taking the change in force divided by the uncorrected slip in the recovery phase of the stress drop, i.e., between points (B) and (C) in Figure S1.

Steady-State Velocities

A description of the fitting used to find the steady-state velocities in Figure 6 (main text) is given in the caption of Figure S3.

Acoustic Emission Localization Algorithm

In order to build an acoustic wave velocity model to locate the acoustic emissions, the seismic velocity in the steel sample holders was found to be 5250 $\frac{\text{m}}{\text{sec}}$ using an oscilloscope. Next, an active survey was performed during an experiment at 20 MPa. Using the

manually-picked arrival times found during this survey and the known locations of the acoustic sensors, an error-minimizing value of 4630 $\frac{\text{m}}{\text{sec}}$ was found for the seismic wave velocity in the norite.

Acoustic emissions are located using a semi-automatic and parallel algorithm presented by Momeni, Liu, and Lecampion (2021). For each experiment, the localization hyper-parameters are tuned in the code. The acoustic emissions are located in 2D and in two steps: the first planar grid of trial hypocenters has a spacing of 5 mm. Then the second 2D grid of trial hypocenters is defined with a spacing of 1 mm around the location obtained in the previous step and with a radius of 10 mm. An example of acoustic emission localization is shown in Figure S13.

For the smoothest sample, we record 3607 events of which 2821 AEs are located, and 1184 of them have localization errors of < 5 mm. For the sample with medium roughness, 3277 events are recorded among which 2718 AEs are located, and 736 AEs have a reasonable location error of < 5 mm. For the test with the roughest rock sample, 1685 events are recorded among which 1604 AEs are located, and 1176 AEs are localized with an error of < 5 mm.

The whole localization for the three mentioned experiments took less than 1 hour on a personal laptop using three 2.6 GHz processors and 16 GB of RAM.

References

Heinze, T., Frank, S., & Wohnlich, S. (2021). FSAT – A fracture surface analysis toolbox in MATLAB to compare 2D and 3D surface measures. *Computers and Geotechnics*, *132*. (https://doi.org/10.1016/j.compgeo.2020.103997)

Momeni, S., Liu, D., & Lecampion, B. (2021). Combining active and passive acoustic

methods to image hydraulic fracture growth in laboratory experiments. *IOP Conference Series: Earth and Environmental Science*, *833*. (https://doi.org/10.1088/1755-1315/833/1/012088)

Violay, M., Giorgetti, C., Cornelio, C., Aeschiman, F., Di Stefano, G., Gastaldo, L., & Wiemer, S. (2021). HighSTEPS: A high strain temperature pressure and speed apparatus to study earthquake mechanics. *Rock Mechanics and Rock Engineering*, *54*, 2039–2052. (https://doi.org/10.1007/s00603-021-02362-w)

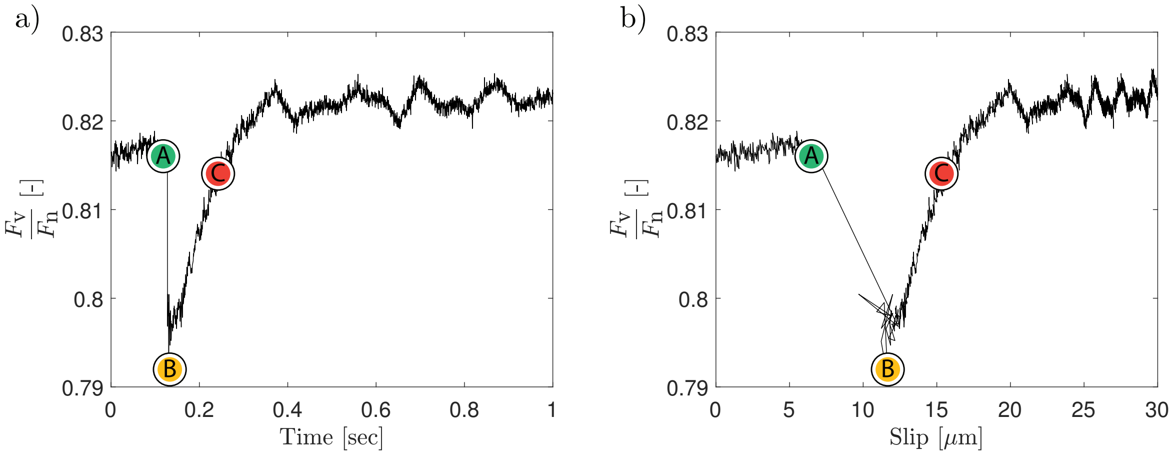

Figure S1. An example of a picked stress drop. The example is taken from a medium roughness sample. The ratio of vertical force to normal load is shown as a function of time (a) and slip (b). (A) represents the start of the stress drop, (B) the minimum value of vertical force, and (C) the recovery. Note that while the slip displays noise near point (B), this generally does not affect the calculation of the maximum slip velocity of the stress drop which is taken between point (A) and point (B). The times and slip listed are not the actual ones from the experiment (they are rescaled to zero at a point just before (A)); this is done to aid legibility.

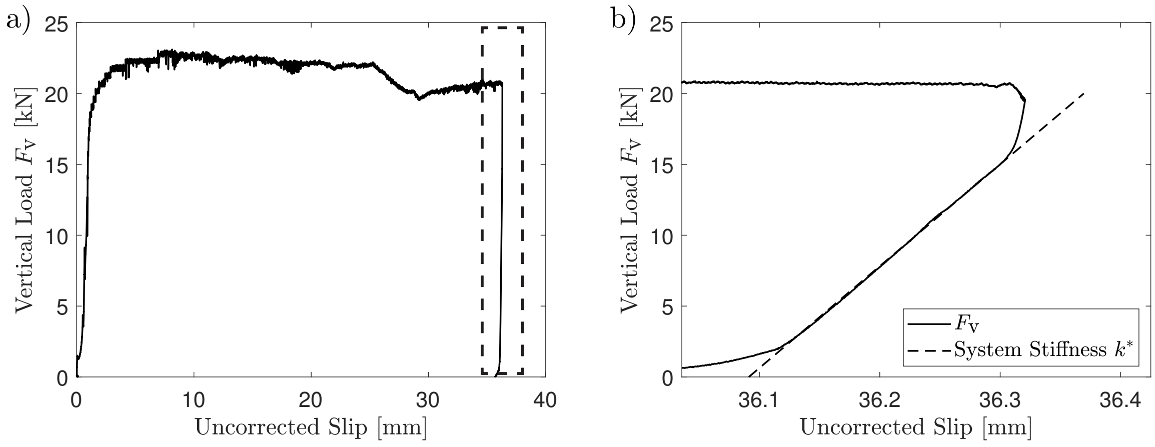

Figure S2. An example of how the system stiffness, k^*, was found. The second unloading stiffness was fit from the plot of vertical load versus uncorrected vertical slip. The example shown here is for a rough sample. The thick dotted box in (a) shows where the zoom used for (b) was taken.

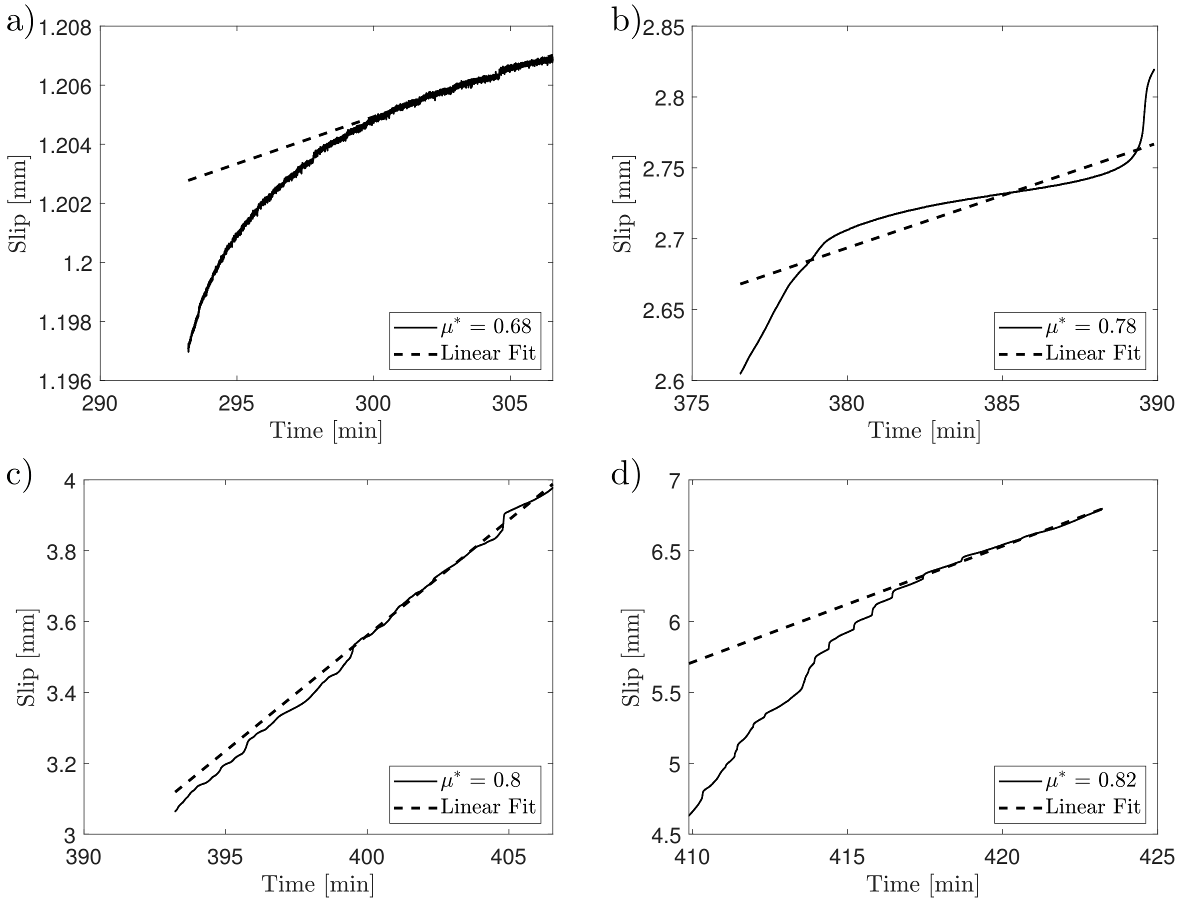

Figure S3. Examples of how the low velocity data points are gathered for Figure 7 (main text). The examples shown are all for a single rough experiment. Each figure represents one load step, with the first and last 100 seconds cut from the step. The slip as a function of time is then linearly fit for the end of the step, with the slope taken as the steady-state velocity of the step. Note that while the fits in (a,c,d) are suitable, due to the lack of a true steady-state, the fit in (b) is poor and results in error in the velocity used in Figure 7 (main text). For each example, μ^*, the apparent friction of the step, is listed in the legend.

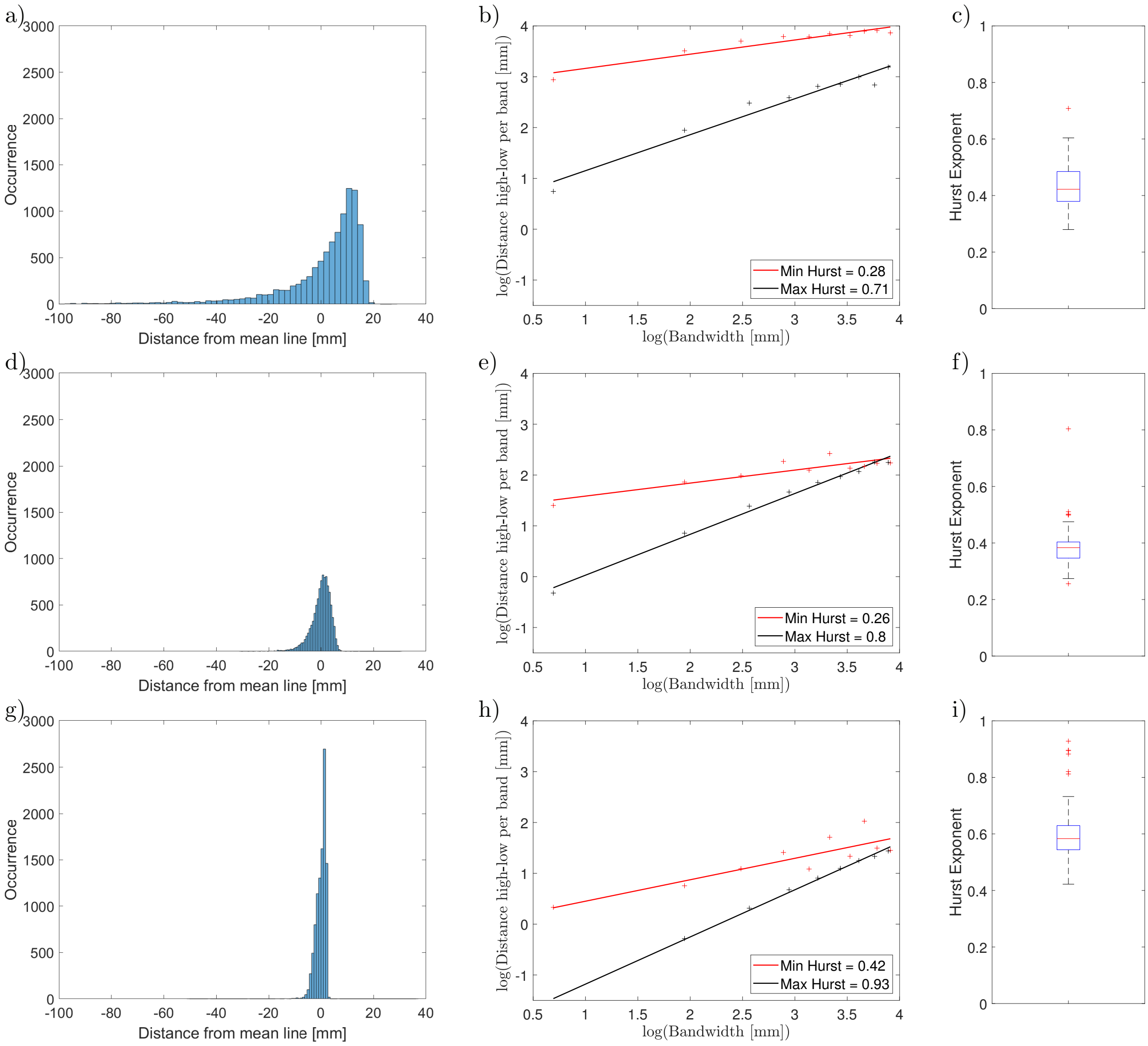

Figure S4. An example of further roughness data for the main suite of experiments. (a,d,g) show the variation from mean height. (b,e,h) show the calculation of the minimum and maximum Hurst exponent. (c,f,i) show a box plot of the possible values found for the Hurst exponent. (a,b,c) represents the rough sample. (d,e,f) represents the medium sample. (g,h,i) represents the smooth sample. All measurements of the Hurst exponent are in the direction of slip. Results were found using the FSAT software from Heinze et al. (2021).

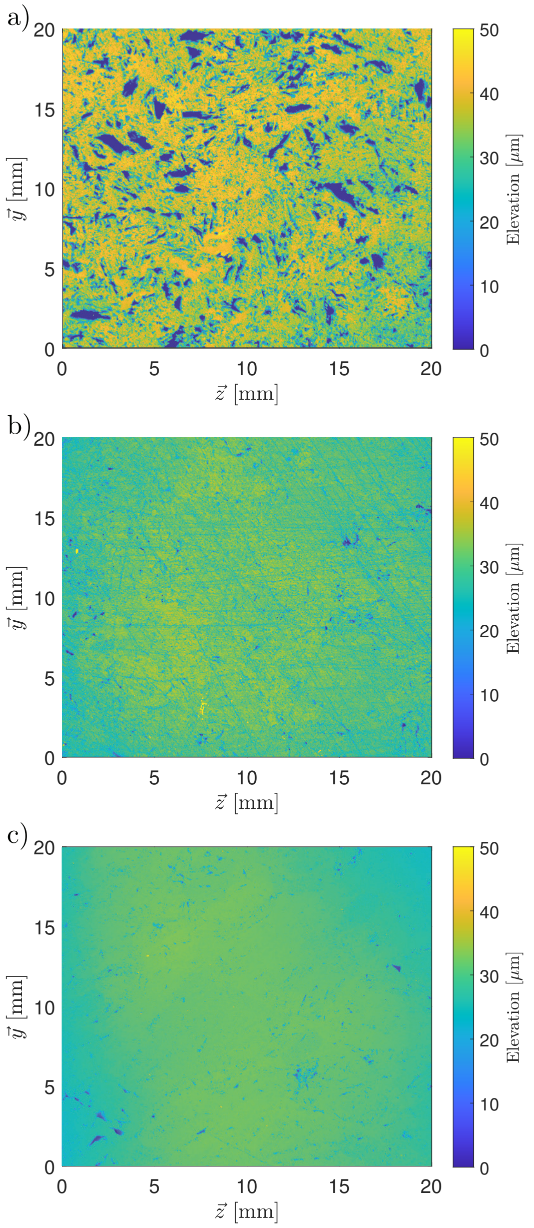

Figure S5. An equivalent to Figure 1(b,c,d) (main text) for the second set of samples. Optical profiler interferometry scans of a portion of the prepared sample surfaces prior to their use during an experiment. a) shows the roughest sample, presenting an R_a of 10.70 μm and an R_q of 16.54 μm, prepared using the milling cutter. b) shows the sample of medium roughness, presenting an R_a of 3.09 μm and an R_q of 4.60 μm, prepared with an 80-grit grinding disc. c) shows the smoothest sample, presenting an R_a of 1.91 μm and an R_q of 2.69 μm, prepared with a 1200-grit grinding disc. The roughness statistics were calculated over an area of 20 mm by 20 mm.

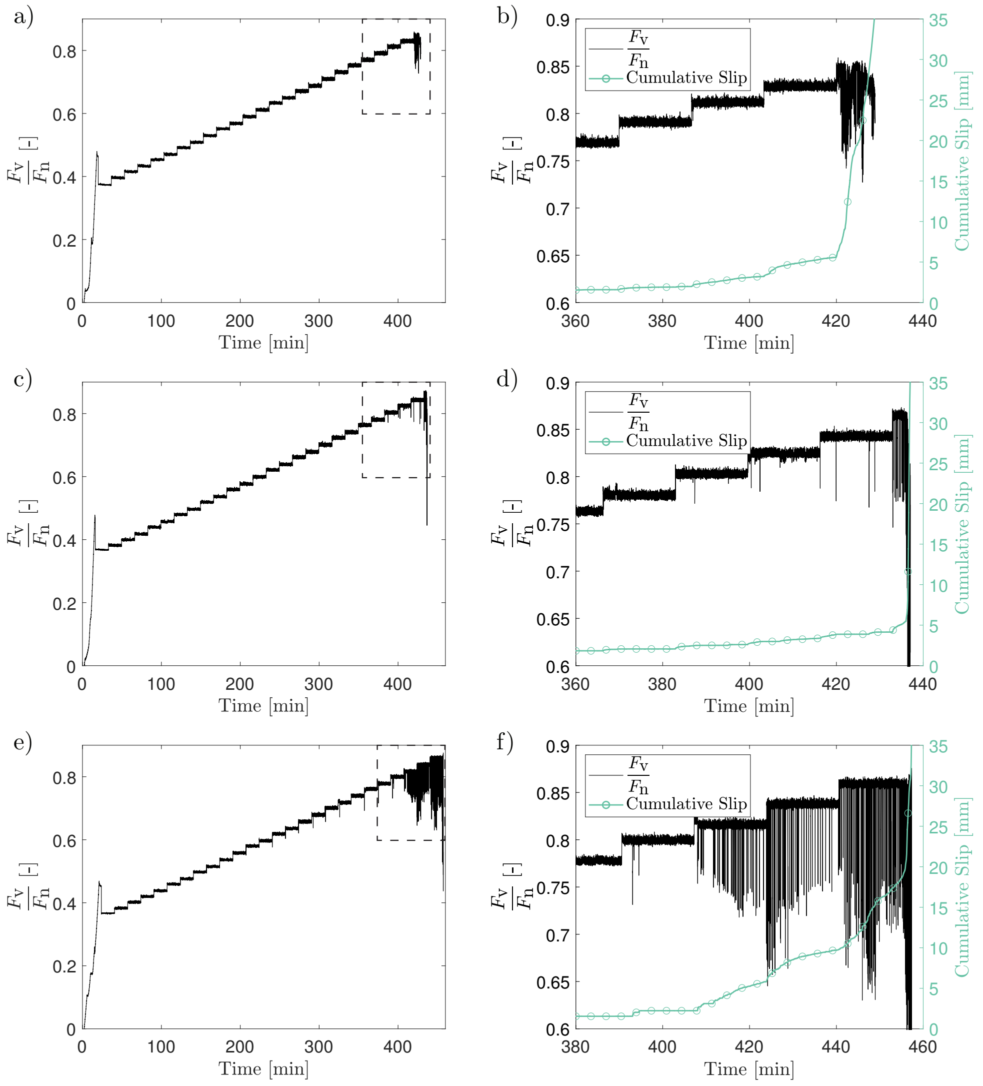

Figure S6. An equivalent to Figure 2 (main text), with all three experiments repeated to show that the results are reproducible. (a,b) represent the roughest sample tested, (c,d) the sample of medium roughness, (e,f) the smoothest sample. (a,c,e) An overview of the development of the ratio between vertical and horizontal force on the sample throughout the experiment. The 1000-second-long vertical force holds can be clearly seen. (b,d,f) show a zoom on the final few steps of each experiment, with an additional line showing the accumulated slip. The stress drops can be seen to be larger and more frequent the smoother the sample is. The smoothest sample also achieved significantly more slip in the steps prior to the final one.

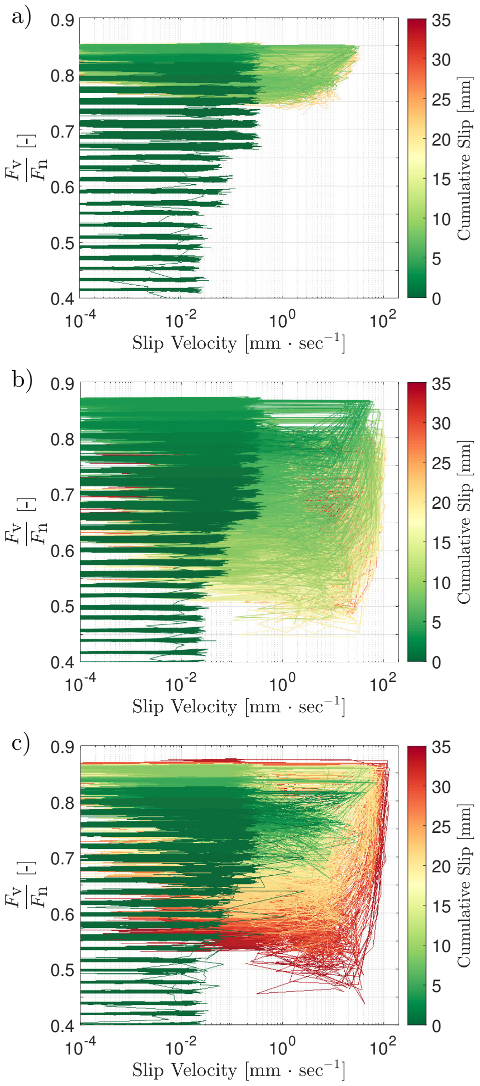

Figure S7. An equivalent to Figure 3 (main text), with all three experiments repeated to show that the results are reproducible. The shear velocity development as the ratio between vertical and horizontal force was increased. The accumulated slip at a given point in the experiment is shown by the color bar. (a) represents the roughest sample tested, (b) the sample of medium roughness, (c) the smoothest sample. The stress drops can be seen to be larger, and the velocity achieved greater, the smoother the sample is. The smoothest sample also achieved significantly more slip in the steps prior to the final one. Note that some of the high velocity slip occurring in (b,c) is occurring during the catastrophic failure at the end of the experiment.

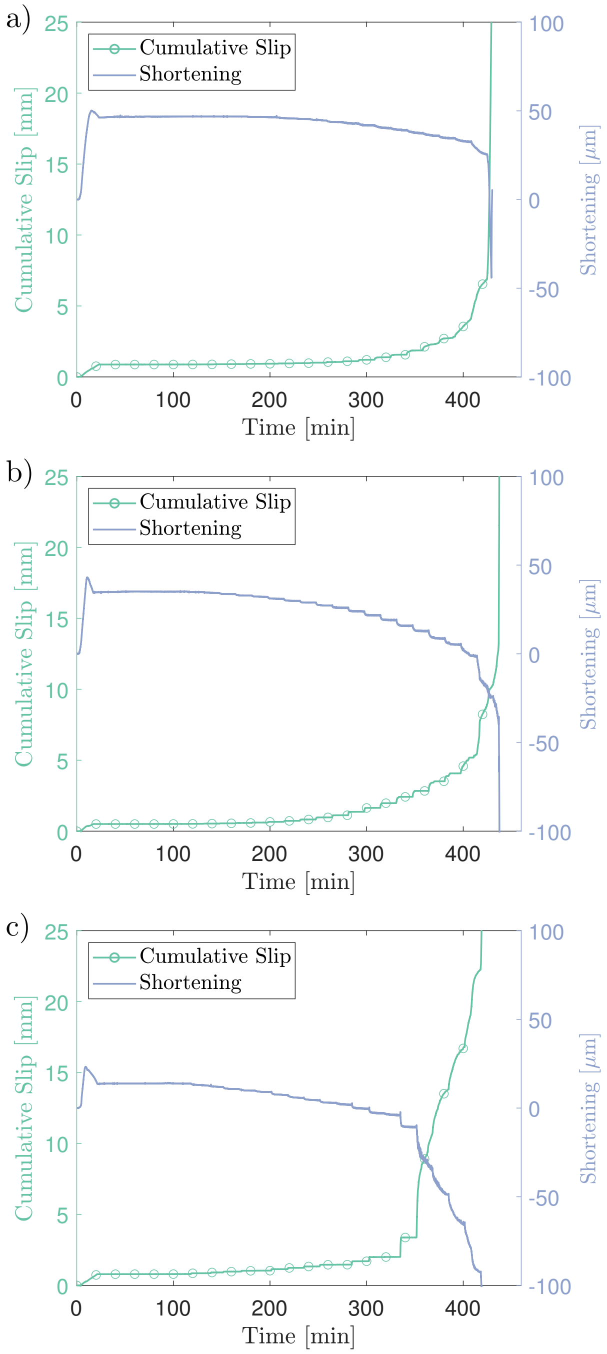

Figure S8. The slip and shortening taking place during each experiment. (a) shows the roughest sample tested, (b) the sample of medium roughness, (c) the smoothest sample. Note that some of the initial slip events, especially for the smooth sample, are related to the sliding events associated with the jumps in stress at the start of each step.

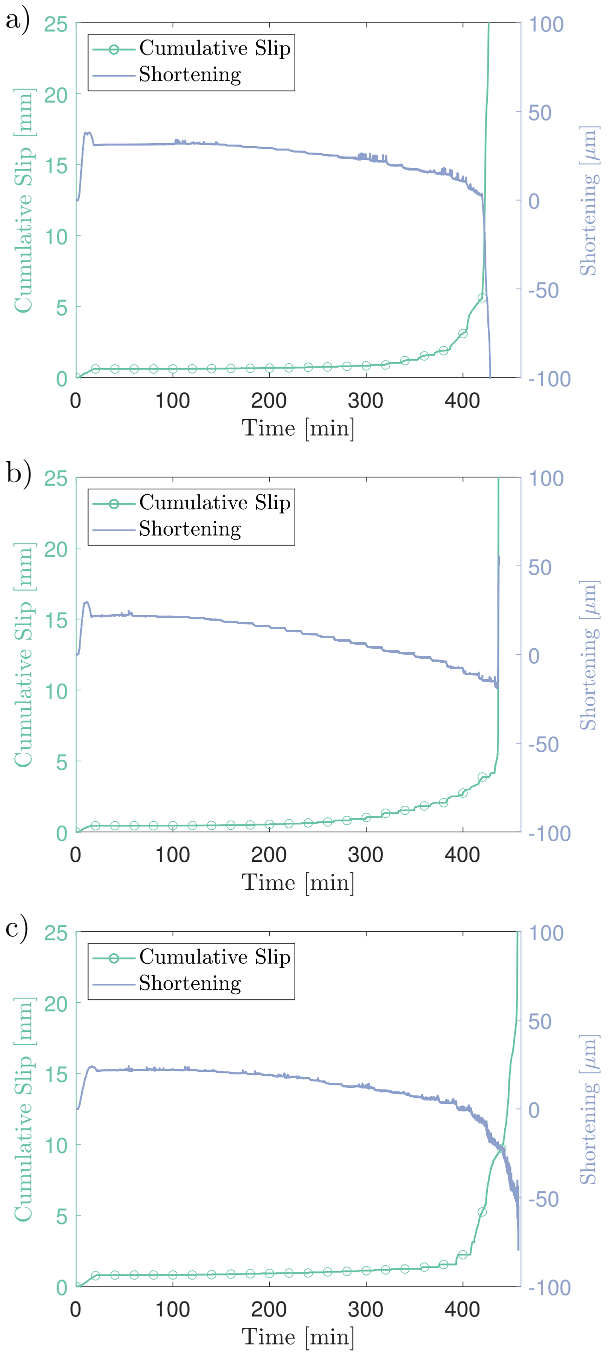

Figure S9. An equivalent to Figure S8 for the second set of samples. The slip and shortening taking place during each experiment. (a) shows the roughest sample tested, (b) the sample of medium roughness, (c) the smoothest sample.

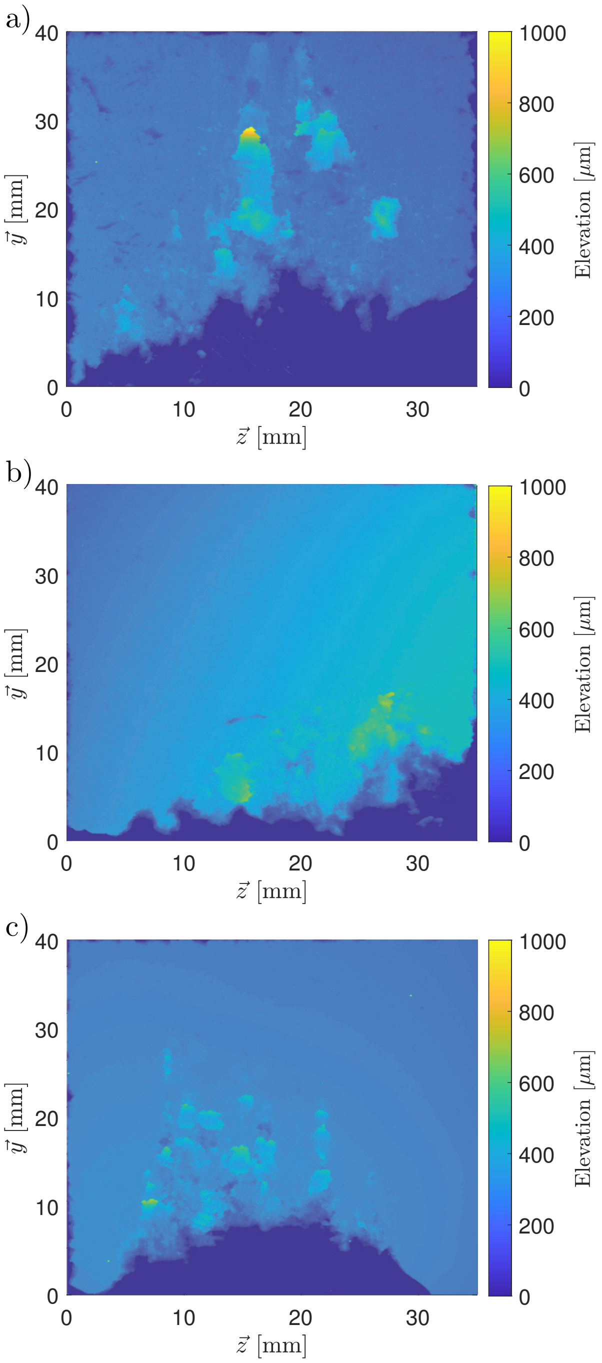

Figure S10. Optical interferometry microscope scans of the second set of the post-mortem sample surfaces. a) shows the roughest sample, b) shows the sample of medium roughness, and c) shows the smoothest sample.

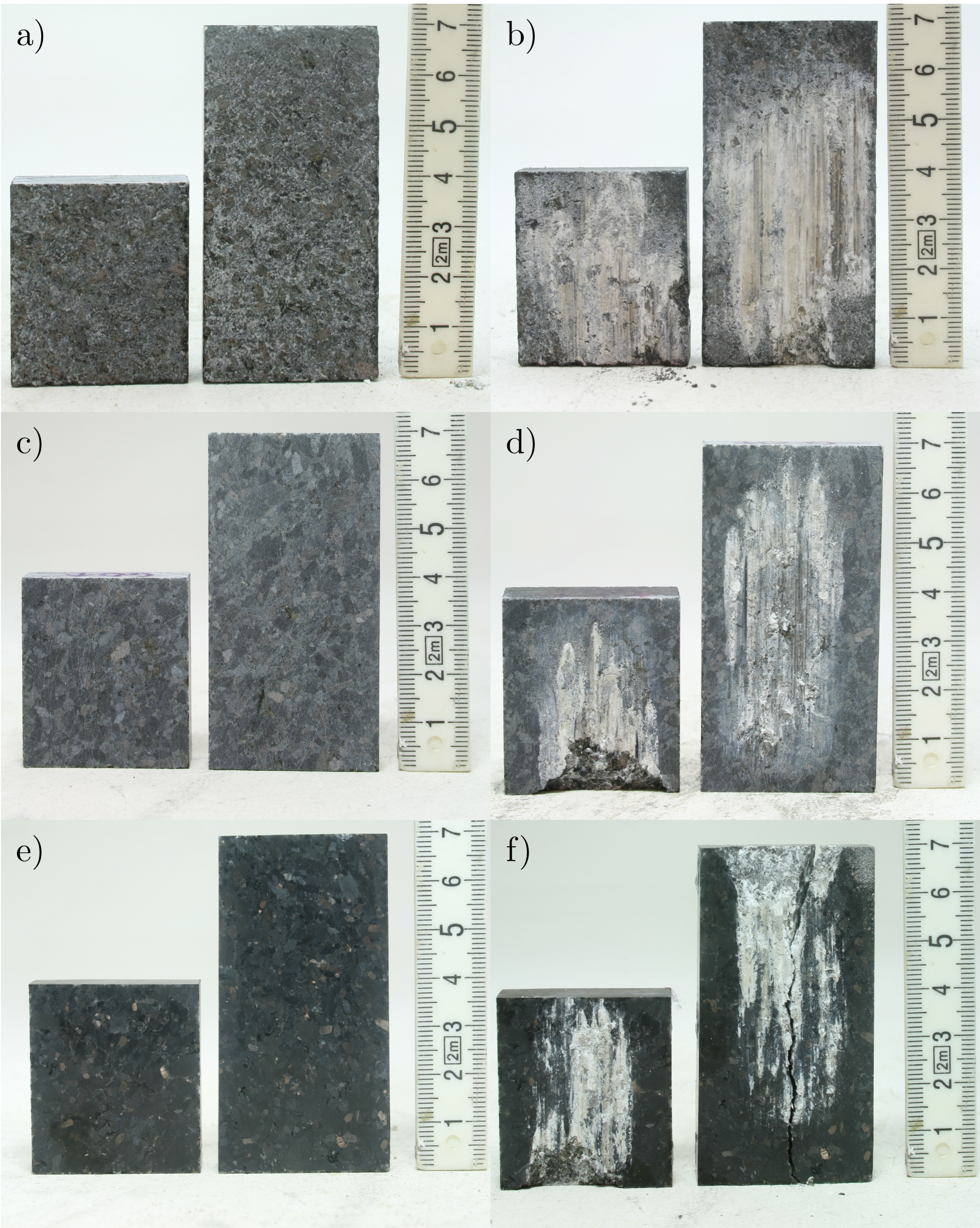

Figure S11. Pre- (a,c,e) and post- (b,d,f) mortem photographs of the sample surfaces of the main suite of experiments. (a,b) show the roughest sample, (c,d) show the medium roughness sample, (e,f) show the smoothest sample.

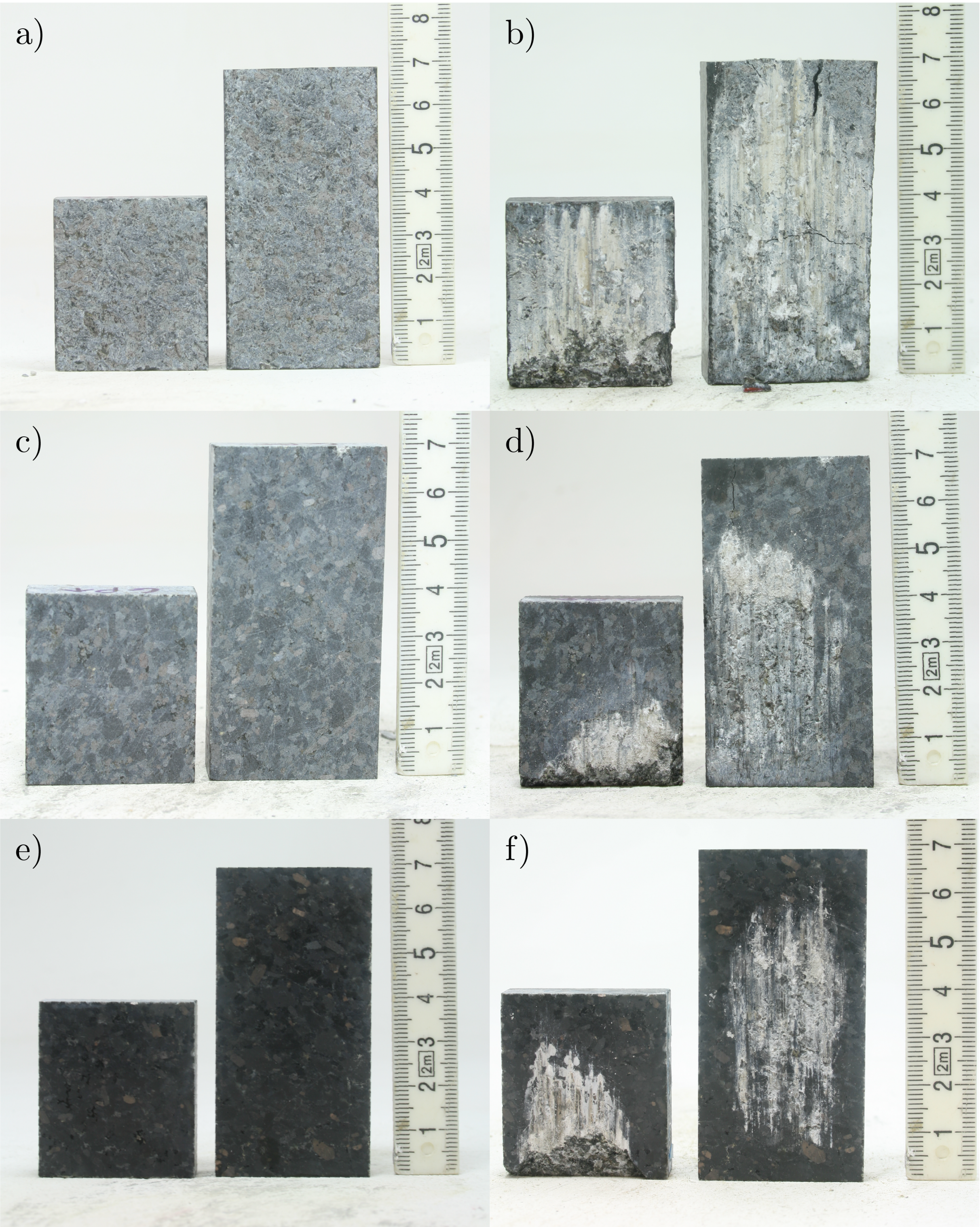

Figure S12. Pre- (a,c,e) and post- (b,d,f) mortem photographs of the sample surfaces of the second set of experiments. (a,b) show the roughest sample, (c,d) show the medium roughness sample, (e,f) show the smoothest sample.

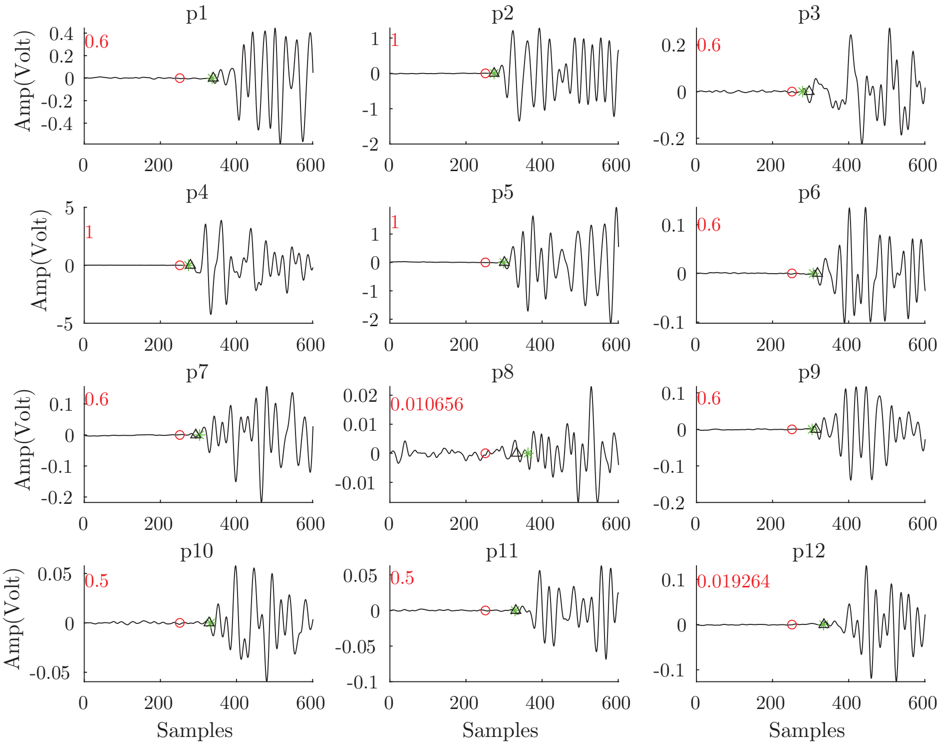

Figure S13. An example of Acoustic Emission localization. The signals are in black, P-wave first arrival time readings in black triangles and green stars, and obtained origin times in red circles. Red numbers on top of each signal represent the weight estimated for that signal and defines its contribution in the localization of the acoustic emission.
